# Wide Range of Mercury Contamination in Chicks of Southern Ocean Seabirds

**DOI:** 10.1371/journal.pone.0054508

**Published:** 2013-01-17

**Authors:** Pierre Blévin, Alice Carravieri, Audrey Jaeger, Olivier Chastel, Paco Bustamante, Yves Cherel

**Affiliations:** 1 Centre d’Etudes Biologiques de Chizé, Centre National de la Recherche Scientifique, Villiers-en-Bois, France; 2 Littoral, Environnement et Sociétés, Centre National de la Recherche Scientifique-Université de La Rochelle, La Rochelle, France; Monash University, Australia

## Abstract

Using top predators as sentinels of the marine environment, Hg contamination was investigated within the large subantarctic seabird community of Kerguelen Islands, a remote area from the poorly known Southern Indian Ocean. Chicks of 21 sympatric seabirds presented a wide range of Hg concentrations, with the highest contaminated species containing ∼102 times more feather Hg than the less contaminated species. Hence, Kerguelen seabirds encompass the whole range of chick feather Hg values that were previously collected worldwide in poorly industrialized localities. Using stable isotopes, the effects of foraging habitats (reflected by δ^13^C) and trophic positions (reflected by δ^15^N) on Hg concentrations were investigated. Species-related Hg variations were highly and positively linked to feather δ^15^N values, thus highlighting the occurrence of efficient Hg biomagnification processes within subantarctic marine trophic webs. By contrast, Hg contamination overall correlated poorly with feeding habitats, because of the pooling of species foraging within different isotopic gradients corresponding to distinct seabird habitats (benthic, pelagic, neritic and oceanic). However, when focusing on oceanic seabirds, Hg concentration was related to feather δ^13^C values, with species feeding in colder waters (lower δ^13^C values) south of Kerguelen Islands being less prone to be contaminated than species feeding in northern warmer waters (higher δ^13^C values). Within the context of continuous increase in global Hg emissions, Kerguelen Islands that are located far away from anthropogenic sources can be considered as an ideal study site to monitor the temporal trend of global Hg contamination. The present work helps selecting some seabird species as sentinels of environmental pollution according to their high Hg concentrations and their contrasted foraging ecology.

## Introduction

Mercury (Hg) is a highly toxic non-essential metal that negatively influences humans and wildlife [1], [2]. In birds, mercury’s adverse effects are far-ranging, with impacts on reproduction being usually the end-point of more direct effects on behaviour, neurology, endocrinology and development [2]. Overall, Hg derives from both natural and anthropogenic sources, but human activities have increased the global amount of Hg cycling around the world by a factor of three to five [3]. Owing to its high volatility and long atmospheric residence time, Hg reaches remote areas through long-range atmospheric transport, thus contaminating oceanic islands and polar and sub-polar regions [4], [5]. Once deposited in aquatic ecosystems, inorganic Hg is subject to biotic reaction (methylation) carried out by microorganisms [6]. Thereafter, methylmercury (Me-Hg), the persistent and highly toxic form of Hg, is assimilated by living organisms *via* food intake, bioaccumulates in individuals and biomagnifies within food webs from lower to higher trophic levels [7]. Hence, top predators have been used to monitor Hg contamination in various aquatic ecosystems [8], with still limited existing information for significant parts of the world ocean, including the southern Indian Ocean [9].

Seabirds are useful bio-indicators of environmental pollution, including Hg contamination because they are long-lived animals that prey at the top of the marine food webs [8], [10]. Feathers are the most attractive avian tissue to sample because, in many cases, they contain most of the Hg body burden and are considered as the main route for Hg elimination [8]. Hg accumulated and stored within soft tissues between moults is transferred and sequestered in the growing feathers and cannot thus be re-incorporated into living tissues. Feather sampling has also the benefit to be non-destructive.

Here, we investigated feather Hg concentrations within the large community of seabirds that breed in Kerguelen Islands, a remote subantarctic archipelago from the Southern Ocean [11]. The seabird assemblage is mainly composed of penguins and Procellariiformes, including the very long-lived and slow-moulting albatrosses that are amongst the most Hg contaminated vertebrate species [12]. Kerguelen seabirds feed on a large diversity of prey (crustaceans, cephalopods and fish) and use contrasted foraging strategies that include benthic and pelagic feeding as well as foraging in neritic and oceanic waters, with the oceanic birds ranging from the warmer subtropical to the colder Antarctic waters (Table 1). The distribution of Hg species in the main oceanic water masses is still poorly documented, with a complete lack of information from Kerguelen waters. However, a recent investigation on Hg speciation within the Southern Ocean revealed a poleward increase in the concentrations of total Hg and Me-Hg in surface waters to some of the highest Me-Hg concentrations so far observed in the open ocean [13]. This Me-Hg increase most likely results from a strong net Hg methylation in the hypoxic zone of the water column in Antarctic waters [13].

**Table 1:** Species, foraging habitats and diets during the chick-rearing period, and durations of the chick rearing period of seabirds at Kerguelen Islands.

Species		Chick rearing	Main foraging habitats		Main prey classes	References
		period (days)	Horizontal	Vertical		
Spheniscidae						
King penguin (*Aptenodytes patagonicus*)	KP	315	oceanic	mesopelagic	mesopelagic fish	[58]
Gentoo penguin (*Pygosce lis papua*)	GP	72	neritic (open sea)	benthic	benthic fish (crustaceans)	[59]
Macaroni penguin (*Eudyptes chrysolophus*)	MP	65	neritic (open sea)/oceanic	epipelagic	crustaceans and fish	Unpublished data
Southern rockhopper penguin (*Eudyptes chrysocome*)	SRP	71	neritic (closed sea)	epipelagic	crustaceans	[60]
Diomedeidae						
Wandering albatross (*Diomedea exulans*)	WA	275	oceanic	sea surface	benthopelagic fish and cephalopods	Unpublished data
Black-browed albatross (*Thalassarche melanophrys*)	BBA	125	neritic (open sea)	sea surface	benthopelagic fish (cephalopods)	[19]
Light-mantled sooty albatross (*Phoebetria palpebrata*)	LMSA	154	oceanic	sea surface	cephalopods (crustaceans, carrion)	[61] a
Procellariidae						
Northern giant petrel (*Macronectes halli*)	NGP	113	on land and at sea	on land, sea surface	carrion/seabirds	[61] a
Grey petrel (*Procellaria cinerea*)	GrP	128	oceanic	sea surface	fish (cephalopods)	Unpublished data
White-chinned petrel (*Procellaria aequinoctialis*)	WCP	96	oceanic	sea surface	fish (cephalopods, crustaceans)	[62]
Great-winged petrel (*Pterodroma macroptera*)	GWP	126	oceanic	sea surface	cephalopods (crustaceans)	[61] a
White-headed petrel (*Pterodroma lessonii*)	WHP	101	oceanic	sea surface	fish (cephalopods)	Unpublished data
Kerguelen petrel (*Aphrodroma brevirostris*)	KeP	60	oceanic	sea surface	crustaceans	[61] a
Blue petrel (*Halobaena caerulea*)	BP	55	oceanic	sea surface	crustaceans (mesopelagic fish)	[49]
Antarctic prion (*Pachyptila desolata*)	AP	50	oceanic	sea surface	crustaceans	[63]
Thin-billed prion (*Pachyptila belcheri*)	TBP	50	oceanic	sea surface	crustaceans	[63]
Pelecanoididae						
Common diving petrel (*Pelecanoides urinatrix*)	CDP	54	neritic (closed sea)	epipelagic	crustaceans	[64]
South-Georgian diving petrel (*Pelecanoides georgicus*)	SGDP	55	oceanic	epipelagic	crustaceans	[64]
Phalacrocoracidae						
Kerguelen shag (*Phalacrocorax verrucosus*)	KS	56	neritic (open sea)	benthic	benthic fish	[65]
Stercorariidae						
Subantarctic skua (*Catharacta antarctica lönnbergi*)	SS	45	on land and at sea	on land, sea surface	small petrels	[47]
Laridae						
Kelp gull (*Larus dominicanus*)	KG	49	on land and at sea	on land, sea surface	carrion/seabirds (limpets)	[66] a

a Stahl and Mougin (1986) and Ridoux (1994) refer to the related Crozet Islands.

In a first descriptive step, Hg was determined in 21 seabird species to assess contamination levels of marine ecosystems within the poorly known southern Indian Ocean. In a second explanatory step, the effect of differences of seabird foraging ecology on feather Hg concentration was tested, because ingestion of food is the main route of Hg exposure in birds [10]. The respective roles of habitats and diets were tested by using the isotopic niche as a proxy of the trophic niche of the species, with the ratios of stable isotopes of carbon (δ^13^C) and nitrogen (δ^15^N) reflecting their foraging habitats and trophic positions, respectively [14]. The isotopic method was already validated in the area, with seabird δ^13^C values indicating their latitudinal foraging grounds and depicting offshore versus inshore consumers [15], [16] and their δ^15^N values increasing with trophic level [17]. Taking into account the species’ foraging ecology, we make the following predictions. Firstly, feeding habitat (δ^13^C) should shape seabird Hg contamination, because Hg is not homogeneously distributed in marine ecosystems. For example, (i) benthic foragers should have relatively high feather Hg concentrations in relation to the substantial production of Me-Hg in coastal marine sediments [6], and (ii) oceanic foragers should be more contaminated in cold than in warm waters, in relation to the latitudinal gradient in the bioavailability of Me-Hg in the Southern Ocean [13]. Secondly, Hg should magnify within subantarctic food webs, which means that seabirds with the highest trophic positions (δ^15^N) should show the highest feather Hg concentrations. Finally, the longer the chick rearing period, the higher the chick feather Hg concentrations should be, because much of the dietary Hg accumulated in soft tissues is mobilized and excreted into growing feathers [18].

Most previous investigations on pollutants in birds were conducted on adults that present some disadvantages related to their foraging areas, moult schedule and migration patterns. Instead, we focused on chicks as indicators and sentinels of contamination [10]. Firstly, young birds that have not yet fledged have obtained all of their food from their parents who forage in the vicinity of the breeding colonies at that time. The chick Hg concentrations and stable isotope signatures thus reflect those of the adult foraging ecology during the chick-rearing period, a period during which feeding information (chick diet and adult foraging grounds) were previously collected using complementary methods (i.e. dietary analysis and bio-logging [19]). Secondly, the integration period is almost identical for Hg and stable isotopes in body feathers of chicks, because (i) Hg accumulated during the chick-rearing period is excreted during the pre-fledging moult, and (ii) feather isotope ratios reflect diet at the time of feather synthesis [20]. Thirdly, since all chick feathers were grown almost simultaneously, both Hg and stable isotopes are fairly homogeneous in the plumage of pre-fledglings [8], [21], while sequential moult in adults induces larger Hg concentrations in the first than in the late growing feathers [22].

## Materials and Methods

### Ethics Statement

Animals were cared for in accordance with the guidelines of the ethics committee of the Institut Polaire Français Paul Emile Victor that approved fieldwork in the present study (Program no. 109, H. Weimerskirch).

### Study Area, Species and Sample Collection

**Figure 1:**
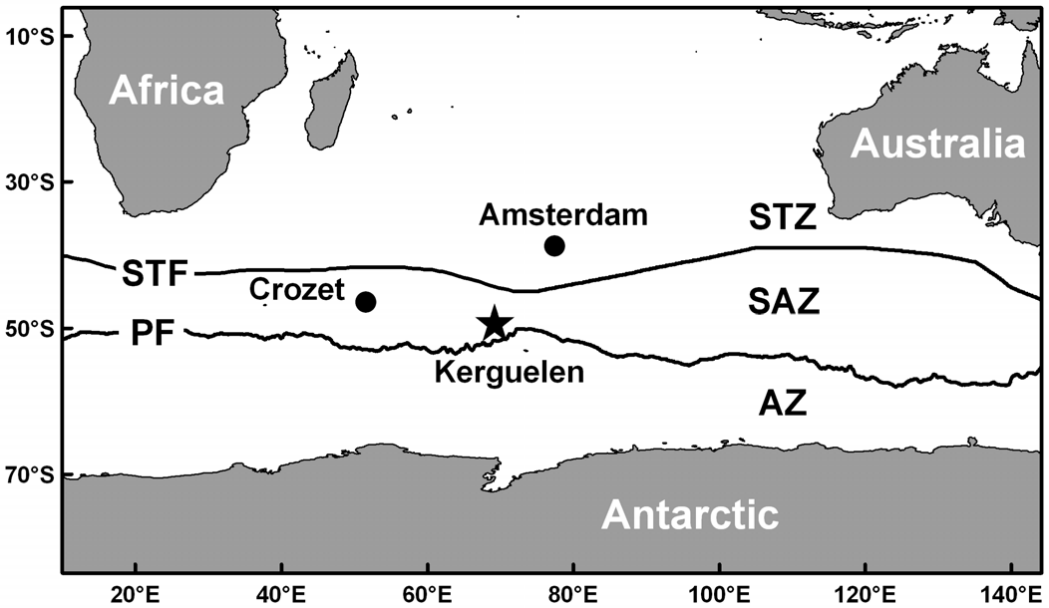
Location of the Kerguelen Islands and of the main oceanic fronts and zones in the southern Indian Ocean. Abbreviations: STF, Subtropical Front; PF, Polar Front; STZ, Subtropical Zone; SAZ, Subantarctic Zone; AZ, Antarctic Zone [23].

Fieldwork was carried out from 2003 to 2012 on Kerguelen archipelago (49°21′S, 70°18′E), which is located in the southern part of the Subantarctic Zone, in the immediate vicinity of the Polar Front [23]. The Southern Ocean was defined as the ocean south the Subtropical Front and the Subantarctic and Antarctic Zones, as the zones between the Subtropical and Polar Fronts, and between the Polar Front and Antarctica, respectively (Fig. 1). Chicks from 21 seabird species belonging to 4 orders and 7 families were sampled, totalizing 280 individuals (n = 7–22 per species). Sampling was conducted at different locations of the archipelago, depending on the species’ breeding sites. Most neritic seabirds were studied in colonies close to the open sea, but two species (the southern rockhopper penguin and common diving petrel) were sampled at Mayes Island that is located within a large bay (closed sea) where the parent birds feed. Body feathers were collected from the lower back of large chicks at fledging, i.e. at the end of the breeding season. Once collected, feathers were stored dry in sealed plastic bags and analysed at the University of La Rochelle, France.

### Sample Analyses

Prior to chemical analysis, all body feathers were cleaned of surface lipids and contaminants using a 2∶1 chloroform: methanol solution for 2 min followed by two successive methanol rinses. They were then cut with scissors into small fragments. A first sub-sample of homogenized feathers was oven dried for 48 hr at 50°C and then analyzed in an advanced Hg analyzer spectrophotometer (Altec AMA 254). Hg determination involved evaporation of Hg by progressive heating until 800°C under oxygen atmosphere for 2′30 min and subsequent amalgamation on a gold trap. The net was heated to liberate the collected Hg that was measured by UV atomic absorption spectrophotometry. Samples were analysed for total Hg, which approximates the amount of Me-Hg since essentially all Hg in feathers is under organic form [24], [25], [26]. All analyses were repeated 2–3 times until having a relative standard deviation <10%. Accuracy was checked using certified reference material (Tort-2 Lobster Hepatopancreas, NRC, Canada; mean 0.27±0.06 µg•g^−1^ dry mass). Our measured values were 0.24±0.01 µg•g^−1^ dry mass, *n* = 56. Blanks were analysed at the beginning of each set of samples and the detection limit of the method was 0.005 µg•g^−1^dry mass.

A second sub-sample of homogenized feathers was weighed (∼0.3 mg) with a microbalance and packed into a tin container. Relative abundance of C and N isotopes were determined with a continuous flow mass spectrometer (Thermo Scientific Delta V Advantage) coupled to an elemental analyzer (Thermo Scientific Flash EA 1112). Results are presented in the δ notation relative to Vienna PeeDee Belemnite and atmospheric N_2_ for δ^13^C and δ^15^N, respectively. Replicate measurements of internal laboratory standards (acetanilide) indicate measurement errors <0.10‰ for both δ^13^C and δ^15^N values.

### Statistical Analyses

Statistical tests were performed using R 2.7.1 [27]. All samples submitted to statistical tests were first checked for normality and homogeneity of variances by means of Shapiro-Wilk and Fisher tests, respectively. Depending on the results, parametric or non-parametric tests were used. A significance level of α <0.05 was used for all tests. Values are means ± SD.

In univariate analyses, correlations between Hg and continuous explanatory variables (feather δ^13^C and δ^15^N values, and the duration of the chick rearing period) were tested using Pearson correlations. In multivariate analyses, the influence of species and continuous explanatory variables on feather Hg concentrations were investigated using General Linear Models (GLMs). Feather Hg concentrations were log transformed and the models were constructed with a normal distribution and an identity link function. Explanatory variables were defined as follows: species as a factor, and feather δ^15^N, δ^13^C values, and the duration of the chick rearing period as covariates. The influence of the sampling year on Hg concentrations was not incorporated in model selection, since, as most species were sampled in only one year (Table 2), the year effect is confounded by the species effect. Noticeably however, two species (the Kerguelen petrel and subantarctic skua) that were sampled twice did not present significant inter-annual differences in feather Hg concentrations (data not shown).

**Table 2:** Chick feather Hg, δ^13^C and δ^15^N values of Kerguelen seabirds.

Species	Years of sampling	*n*	Total Hg (µg•g^−1^ dry mass)	δ^13^C (‰)	δ^15^N (‰)
South Georgian diving petrel	2012	16	0.05±0.01 (0.04–0.08)	**−**21.3±0.3	8.8±0.3
Common diving petrel	2003	17	0.11±0.02 (0.07–0.15)	**−**17.0±0.5	12.1±0.4
Antarctic prion	2008	10	0.21±0.05 (0.16–0.31)	**−**21.5±0.5	9.3±0.4
Thin-billed prion	2003	9	0.22±0.09 (0.12–0.40)	**−**21.5±0.5	9.1±0.4
Southern rockhopper penguin	2007	12	0.27±0.06 (0.20–0.37)	**−**15.3±0.4	11.5±0.4
Macaroni penguin	2007	12	0.36±0.07 (0.25–0.52)	**−**18.3±0.5	10.0±0.5
Kelp gull	2011	7	0.73±0.38 (0.40–1.38)	**−**12.8±0.7	13.4±1.0
Kerguelen petrel	2009, 2010	18	0.78±0.17 (0.51–1.20)	**−**22.1±0.5	11.7±0.5
Blue petrel	2003	13	0.84±0.18 (0.58–1.14)	**−**21.8±0.5	9.8±0.5
King penguin	2006	12	1.12±0.16 (0.83–1.50)	**−**21.6±0.3	10.6±0.3
White-headed petrel	2003	10	1.54±0.34 (1.07–1.99)	**−**22.0±0.5	12.2±0.2
Great-winged petrel	2005	10	1.64±0.48 (0.96–2.68)	**−**20.0±0.4	12.9±0.4
White-chinned petrel	2005	14	1.82±0.51 (1.13–2.76)	**−**22.2±0.7	11.3±0.8
Kerguelen shag	2006	10	2.21±1.06 (1.35–4.64)	**−**13.8±1.0	14.0±0.6
Gentoo penguin	2006	12	2.45±0.67 (1.14–3.66)	**−**16.5±1.2	12.4±0.8
Light-mantled sooty albatross	2005	15	2.46±0.67 (1.56–3.69)	**−**21.0±0.4	12.6±0.4
Black-browed albatross	2005	18	2.58±0.59 (1.54–3.70)	**−**18.5±0.8	12.9±0.5
Grey petrel	2005	16	3.16±1.21 (1.59–5.70)	**−**19.9±0.6	13.6±0.4
Wandering albatross	2005	15	4.45±1.60 (2.19–8.43)	**−**19.3±0.4	14.2±0.4
Subantarctic skua	2005, 2010	22	5.15±1.56 (2.40–7.93)	**−**21.8±0.4	10.8±0.3
Northern giant petrel	2005	12	5.31±1.12 (4.06–7.94)	**−**19.2±1.2	13.4±0.8

Species were deliberately ranked by increasing Hg concentrations and not in taxonomic order. Values are means ± SD with ranges in parentheses for Hg.

Biologically relevant models were constructed by incorporating the different variables and their interactions. Continuous variables that were significantly correlated were not included in the same models. The most parsimonious models were selected according to the bias-adjusted Akaïke’s Information Criterion (AICc), which is a small-sample bias adjustment [28], [29]. As a general guideline, if AICc values differ by more than 2, the model with the lowest AICc value is the most accurate, whereas models with AICc values differing by less than 2 are fairly similar in their ability to describe the data, and the model including the least number of parameters (the simplest) is the most accurate [30]. The likelihood of a model, referred to as the Akaïke weight (*w_i_*) was estimated following Johnson and Omland [31] (Table 3). The *w_i_* can be interpreted as approximate probabilities that the model *i* is the best one for the observed data, given the candidate set of models. Model fit was assessed by a chi-square goodness-of-fit test [32], and residuals were checked for normality using Shapiro-Wilk test and Q-Q plot. The coefficient of determination *R^2^adj* was calculated for each model [31] (Table 3).

**Table 3:** AICc model ranking for feather Hg concentrations within the Kerguelen seabird community (see text for details).

Models	AICc	ΔAICc^a^	*w_i_* b	*R^2^adj*	*gdf*
species+δ^15^N	78.79	0.00	0.999	0.96	1.00
species+δ^13^C+species* δ^13^C	97.08	18.29	<0.001	0.96	1.00
species+δ^15^N+species* δ^15^N	99.36	20.57	<0.001	0.96	1.00
species+CRP+ species* CRP	110.12	31.33	<0.001	0.96	1.00
species+CRP	110.12	31.33	<0.001	0.96	1.00
species	110.12	31.33	<0.001	0.96	1.00
species+δ^13^C	110.20	31.41	<0.001	0.96	1.00
δ^15^N	841.16	762.37	<0.001	0.37	0.03
CRP	930.02	851.23	<0.001	0.14	<0.001
null	971.35	892.56	<0.001	0.00	<0.001
δ^13^C	972.11	893.32	<0.001	<0.01	<0.001

Abbreviations: AICc, bias-adjusted Akaike’s Information Criteria values;

*w_i_*, AICc weights; *R^2^adj*, R-squared adjusted; *gdf*, goodness-of-fit; CRP, duration of the chick rearing period.

a Scaled ΔAICc; ΔAICc = 0.00 is interpreted as the best fit to the data among the models.

b Weight of evidence interpreted as a proportion. Weights across all models sum to 1.00.

## Results

Univariate analysis showed that total Hg contamination varied widely within the seabird community, with chick feather Hg concentrations differing significantly between species (Kruskal-Wallis, *H = *260.23, *P*<0.001, *n* = 21). Mean Hg concentrations ranged from 0.05±0.01 to 5.31±1.12 µg•g^−1^ dry mass in South-Georgian diving-petrels and northern giant petrels, respectively (Table 2). The lowest chick feather Hg concentration occurred in a common diving-petrel and the highest in a wandering albatross (0.04 and 8.43 µg•g^−1^, respectively).

**Figure 2:**
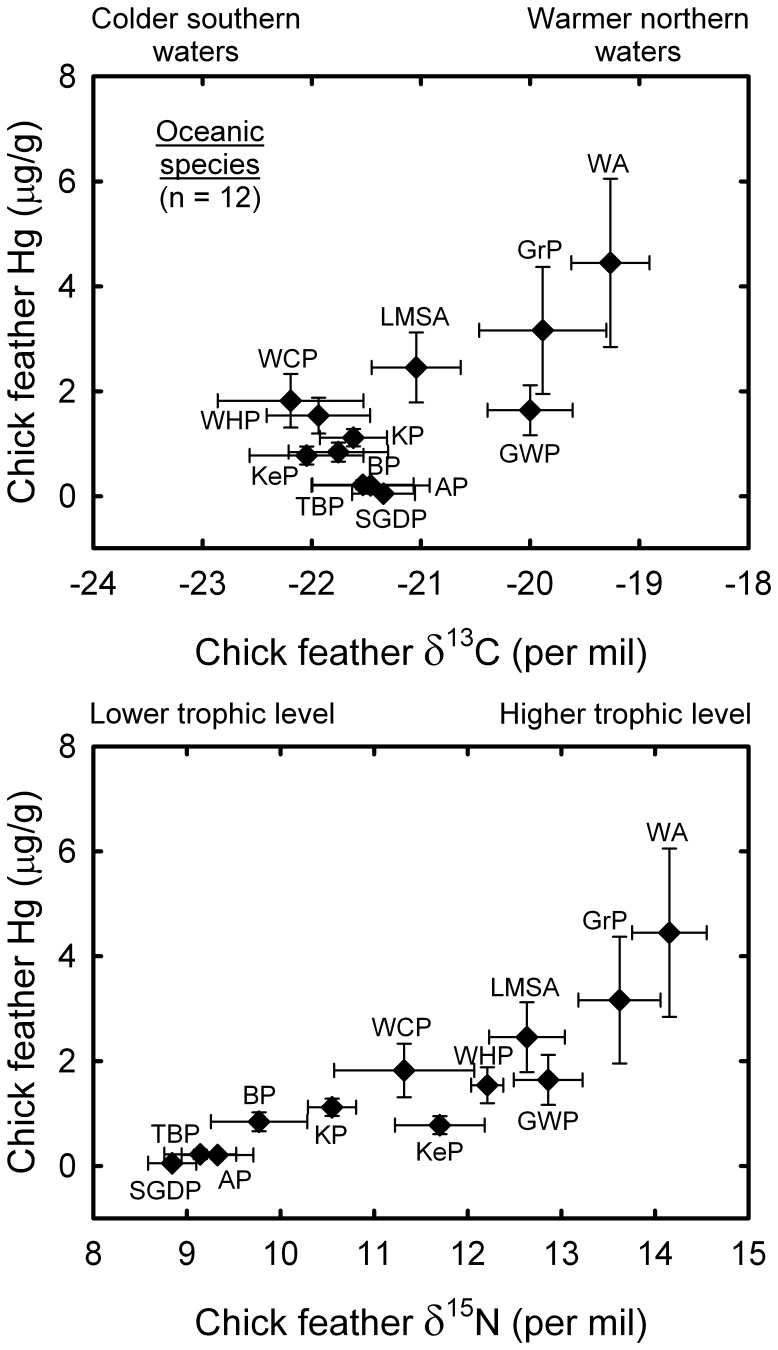
Relationship between chick feather Hg concentrations (means ± SD; µg•g^−1^ dry mass) and (a) foraging habitat (chick feather δ^13^C) and (b) trophic position (chick feather δ^15^N) of oceanic species. Filled diamonds and empty circles refer to oceanic and other species, respectively. See Table 1 for species abbreviations.

No overall significant correlation between feather Hg and δ^13^C values was found (Pearson correlation, *r* = **−**0.08, *t* = **−**1.31, *P* = 0.19, *n* = 280), while feather Hg concentration was highly significantly and positively correlated with δ^15^N values (*r* = 0.48, *t* = 9.19, *P*<0.001). Feather Hg concentration was also significantly and positively related to the duration of the chick rearing period (*r* = 0.26, *t* = 4.54, *P*<0.001). Furthermore, feather δ^15^N and δ^13^C values were positively correlated (*r* = 0.63, *t* = 10.24, *P*<0.001), and they were significantly related to the duration of the chick rearing period (*r* = **−**0.12 and 0.35, *t* = **−**2.10 and 6.33, *p* = 0.037 and <0.001 for δ^13^C and δ^15^N values, respectively). When focusing on oceanic seabirds (12 species and 158 individuals) a highly significant and positive correlation was found between feather Hg and both δ^13^C (*r* = 0.61, *t* = 9.68, *p*<0.001) and δ^15^N values (*r* = 0.80, *t* = 16.90, *P*<0.001) (Fig. 2).

In multivariate analyses, the most parsimonious GLM model selected by AICc values included the effects of species and δ^15^N (Table 3) in explaining Hg concentrations in body feathers. Indeed, Hg concentrations differed significantly among species (*F*20, 258 = 217, *P*<0.001) and were significantly and positively related to their δ^15^N values (*F*1, 278 = 2776, *P*<0.001). The second-ranked model included an effect of species, δ^13^C and their interaction, but it had a low likelihood when compared to the first-ranked model (Table 3). Models including the duration of the chick rearing period had also a low likelihood, thus explaining poorly feather Hg concentrations when compared to species and feather δ^15^N values.

## Discussion

To the best of our knowledge, this study is the first to investigate Hg contamination in such a large number of sympatric seabirds. It completes the few works previously conducted in the southern Atlantic [33], [34] and the southern Pacific Oceans [35], [36], thus partly filling the gap of knowledge from the southern Indian Ocean sector [9]. The study adds a substantial number of seabird chicks that were either not previously investigated (n = 16) or inadequately sampled (n = 4) [35], with only data from wandering albatross [37] being available in the scientific literature (Table 4).

**Table 4:** An overall synthesis of Hg concentrations (means ± SD with ranges in parentheses; µg•g^−1^ dry mass) in body feathers of seabird chicks.

Species	Location	n	Total Hg	References
Spheniscidae				
Adelie penguin (*Pygoscelis adeliae*)	Terra Nova Bay (Antarctica)	11	0.37±0.15	[67]
Diomedeidae				
Wandering albatross (*Diomedea exulans*)	Bird Island (South Georgia)	10	3.31±0.68	[37]
Black-footed albatross (*Phoebastria nigripes*)	Midway Atoll	17	5.57±0.36^a^	[40], [68]
Laysan albatross (*Phoebastria immutabilis*)	Midway Atoll	35	2.15±0.12^a^	[40], [68]
		15	1.95±0.15^a^	[69]
Procellariidae				
Barau’s petrel (*Pterodroma baraui*)	La Réunion	32	0.30±0.07	[41]
Bonin petrel (*Pterodroma hypoleuca*)	Midway Atoll	20	3.87±0.32^a^	[40], [70]
Audubon’s shearwater (*Puffinus lherminieri*)	La Réunion	38	0.07±0.01	[41]
	Aride Island (Seychelles)	10, 8	0.15±0.03, 0.27±0.06	[38]
Pink-footed shearwater (*Puffinus creatopus*)	Mocha Island (Chile)	8	0.36±0.10	[71]
Sooty shearwater (*Puffinus griseus*)	Humboldt Current (Peru)	14	0.72±0.35	[72]
	New Zealand Region	4	0.8±0.8	[35]
Wedge-tailed shearwater (*Puffinus pacificus*)	Aride Island (Seychelles)	10	0.39±0.05	[38]
Cory’s shearwater (*Calonectris diomedea*)	Azores	14	0.87±0.10^a^ (0.33–1.5)	[73]
		30	0.7±0.2	[74]
	Berlengas (Portugal)	25	1.1±0.3	[74]
Hydrobatidae				
Leach’s storm-petrel (*Oceanodroma leucorhoa*)	New Brunswick (Canada)	20	1.42 (0.90–2.22)^b^	[75]
Phaethontidae				
Red-tailed tropicbird (*Phaethon rubricauda*)	Midway Atoll	12	2.51±0.28^a^	[40], [70]
White-tailed tropicbird (*Phaethon lepturus*)	La Réunion	16	0.29±0.02	[41]
	Aride Island (Seychelles)	10, 10	0.52±0.11, 0.70±0.10	[38]
Phalacrocoracidae				
European shag (*Phalacrocorax aristotelis*)	Atlantic sector (Spain)	20, 12	from 0.54±0.19 to 1.07±0.38	[76]
	Cantabrian sector (Spain)	15, 10	3.09±1.40, 5.09±1.82	[76]
Stercorariidae				
Great skua (*Catharacta skua*)	Foula (Shetland)	40	1.3±0.4	[54]
		28	1.22±0.38	[77]
		29	2.16±1.15	[53]
	St Kilda (Outer Hebrides)	22	5.37±1.29	[53]
Arctic skua (*Stercorarius parasiticus*)	Foula (Shetland)	30	0.46±0.22	[77]
Laridae				
Audoin’s gull (*Larus audouinii*)	Dodecanese (Greece)	20, 10	from 0.94±0.27 (0.61–1.46) to 1.71±0.48 (0.32–2.55)	[78]
	Cyclade (Greece)	20, 10	from 1.42±0.29 (0.88–2.04) to 2.02±0.38 (1.13–2.45)	[78]
	Kythera (Greece)	8	1.20±0.33 (0.76–1.77)	[78]
	Ebro Delta (Western Mediterranean)	39	5.09 (4.68–5.54)^d^	[79]
	Alboran Island (Western Mediterranean)	15	3.87 (3.28–4.57)^d^	[79]
	Chafarinas Islands (Western Mediterranean)	12	3.17 (2.31–4.35)^d^	[79]
Black-headed gull (*Larus ridibundus*)	German North Sea	36	0.88±0.53 (0.14–2.11), 0.94±0.45 (0.10–2.07)	[80]
Common gull (*Larus canus*)	Elbe estuary (German North Sea)	12	2.24±1.84	[81]
	Jade Bay (German North Sea)	11	1.40±0.37	[81]
Franklin’s gull (Larus pipixcan)	Interior U.S.A.	≥79	0.80±0.06a	[82]
	Minnesota	15	0.31±0.11a	[83]
Glaucous-winged gull (*Larus glaucescens*)	Aleutian Islands	36	1.98±0.18^a^	[84]
Herring gull (*Larus argentatus*)	New York	15, 20, 15	0.81±0.12, 1.80±0.11, 2.83±0.27^a^	[85], [86]
	New Jersey	14, 15	1.76±0.35, 2.58±0.23^a^	[86]
	Virginia	15	0.76±0.11^a^	[86]
	German North Sea	38	5.88±4.90 (0.78–27.14)	[87], [88]
		39	1.27±0.60 (0.47–2.98), 1.31±0.62 (0.49–2.89)	[80]
	Shetland	12	2.24±0.83 (1.04–4.12)	[88]
Red-billed gull (*Larus novaehollandiae*)	Kaikoura Peninsula (New Zealand)	27	2.02±1.16	[89]
Yellow-legged gull (*Larus michahellis atlantis*)	Azores	34	2.3±1.0	[74]
	Madeira	22	2.6±0.8	[74]
	Berlengas (Portugal)	28	2.4±0.5	[74]
Kittiwake (*Rissa tridactyla*)	Foula (Shetland)	26	0.37±0.12	[77]
	Shetland	9	0.49±0.28 (0.26–1.03)	[88]
	German North Sea	13	2.65±0.61 (1.61–3.64)	[88]
	Northeast Norway	27	0.55±0.10	[89]
Sternidae				
Arctic tern (*Sterna paradisaea*)	Foula (Shetland)	15	0.69±0.14	[77]
Common tern (*Sterna hirundo*)	Bird island (Massachusetts)	21	3.1±0.2^a^	[90]
		15	4.2±3.1	[91]
	Long Island (New York)	16	1.4±0.6 (0.6–2.6)	[92]
		14, 21	2.01±0.25, 2.61±2.55^a^	[93]
	German North Sea	21	6.14±4.33 (1.51–18.40)	[87]
		13	3.00±0.50 (1.97–3.74), 3.26±0.70 (2.41–4.91)	[80]
		27	12.89±6.90 (1.51–70.00)	[88]
	Elbe estuary (German North Sea)	4	36.4±18.9 (21.7–62.9)	[88]
	Jadebusen (German North Sea)	9	3.8±0.7 (2.9–5.1)	[88]
	East Scotland	19	1.80±0.79 (0.92–3.11)	[88]
	Shetland	12	1.40±0.72 (0.84–2.95)	[88]
	Azores	10, 19	from 1.1±0.4 to 1.5±0.4	[74]
Forster’s tern (*Sterna forsteri*)	San Francisco Bay	89	6.44±0.28^d^	[94]
Little tern (*Sterna albifrons*)	Portugal	168	4.07±1.42	[95]
	Vaia (Portugal)	12, 10	4.40±1.31, 4.67±1.38	[96]
Roseate tern (*Sterna dougallii*)	Azores	19, 14	0.8±0.2, 1.1±0.2	[74]
	Aride Island (Seychelles)	12	0.69±0.32	[97]
White tern (*Gygis alba*)	Aride Island (Seychelles)	10, 10	0.21±0.03, 0.40±0.05	[38]
	Midway Atoll	7	1.65±0.18^a^	[40], [98]
Sooty tern (*Onychoprion fuscata*)	Lys (Glorieuses)	32	0.05±0.03	[41]
	Aride Island (Seychelles)	10	0.26±0.05	[38]
	Hawaii	16	0.16±0.02^a^	[98]
Brown noddy (*Anous stolidus*)	Aride Island (Seychelles)	10, 10	0.27±0.05, 0.37±0.06	[38]
	Hawaii	20	0.07±0.003^a^	[99], [100]
Lesser noddy (*Anous tenuirostris*)	Aride Island (Seychelles)	10, 5	0.17±0.03, 0.41±0.17	[38]
Alcidae				
Razorbill (*Alca torda*)	New Brunswick (Canada)	16	1.40 (0.86–2.29)	[75]
Common murre (*Uria aalge*)	New Brunswick (Canada)	9	1.14 (0.59–2.18)^b^	[75]
Atlantic puffin (*Fratercula arctica*)	New Brunswick (Canada)	17	1.00 (0.27–0.73)^b^	[75]

Down Hg values and studies with too low numbers of sampled chicks (n <4) were excluded.

a Values are means ± SE.

b Values are estimated marginal means with 95% confidence limits in parentheses.

c Median value.

d Values are geometric means with 95% confidence limits in parentheses.

Statistical analyses pointed out the important effect of species, feeding habits (δ^15^N) and foraging habitats (δ^13^C) on chick feather Hg concentrations, which, by contrast, are little explained by the duration of the chick rearing period. However, univariate analysis showed a positive relationship between Hg contamination and the duration of the chick- rearing period, the most likely explanation being that assimilated Hg accumulates during chick growth over weeks and months and is ultimately excreted in newly grown feathers at the end of the period [18]. The positive co-variation between the duration of the chick rearing period and δ^15^N likely results from the longer chick rearing period of large seabirds (*e.g.* albatrosses) that feed at higher trophic positions than smaller species with shorter growth period (*e.g.* petrels).

### Feather Hg Concentrations: Comparison with Other Species and Areas

Kerguelen seabird chicks presented a wide range of Hg concentrations, with the highest contaminated species containing ∼102 times (two orders of magnitude) more feather Hg than the less contaminated species. Both the lowest and highest Hg values occurred in flying birds, with contamination levels of the flightless penguins ranging from low to intermediate values. No other feather Hg concentrations are available from Kerguelen seabirds, but a preliminary analysis conducted on internal tissues of adults of five species of zooplankton-eating petrels [9] ranked the species in the same decreasing order than in the present study, with blue petrels containing more Hg than prions and diving petrels. In the same way, the decreasing order is roughly the same in the only other comparable investigations that were conducted on seabirds breeding in the southern Atlantic Ocean [33], [37]. Feather Hg was higher in albatrosses and giant petrels, intermediate in the white-chinned petrel and lower in the blue petrel, prions and diving petrels. Hg concentrations were however much higher in south Atlantic Procellariiformes than in the present study [33], with the most likely explanation being that work was conducted on adult birds, not on chicks. Indeed, adult birds have consistently higher feather Hg concentrations than their chicks [38]. Adults have a longer period to assimilate and accumulate metals from their food between two successive moults, whereas chicks only have the several weeks (to months) of the chick-rearing period [39].

A review of the existing literature on seabird chicks (Table 4) shows that mean feather Hg concentration can reach very high values in acutely polluted areas (up to 36.4 µg•g^−1^ for common terns in the German North Sea). Elsewhere, however, Hg concentration ranges from 0.05 to 5.6 µg•g^−1^ (the sooty tern and black-footed albatross, respectively). This range is remarkably similar to that from the present investigation, indicating that seabirds from only one location (Kerguelen) encompass the whole range of values that were collected worldwide in poorly industrialized areas. Consequently, comparison of Hg levels between distant locations using seabirds as sentinels of environmental contamination necessitates collecting feathers from many species, because a small subset could not be fully representative of the whole seabird assemblage, and thus of the surrounding marine environment.

At the species level, chick feather Hg concentrations from Kerguelen seabirds fall within the concentration range reported for similar species elsewhere. The wandering albatross from South Georgia [37], presented identical Hg concentrations than chicks from Kerguelen, and the taxonomically closely-related but spatially distant Scottish great skua and Kerguelen subantarctic skua showed almost similar feather Hg concentrations, depending on breeding colonies (Table 4). At the community level, only two previous investigations on chick Hg contamination included more than five sympatric species and both were conducted in tropical waters of the Pacific (Midway Atoll, six species [40]) and the Indian Oceans (the Seychelles, seven species [38]). Based on feather Hg concentrations, the Kerguelen seabird community compares well with the Midway Atoll assemblage (0.34–5.57 µg•g^−1^) that also includes albatrosses, i.e. the species group that contains the highest feather Hg concentrations among seabirds [12] (Table 4). By contrast (and excluding albatross data), chick feather Hg concentrations were overall higher in Kerguelen species than in seabirds from the Seychelles (0.15–0.70 µg•g^−1^) and La Réunion (0.07–0.42 µg•g^−1^ [41]), thus suggesting higher Me-Hg bioavailability in subantarctic than in tropical waters of the Indian Ocean. The hypothesis remains to be investigated because there is a paucity of information on mercury speciations in marine waters, and more specifically, on the sources of Me-Hg to marine consumers, including seabirds and their prey [6], [13].

### Potential Adverse Effect

**Figure 3:**
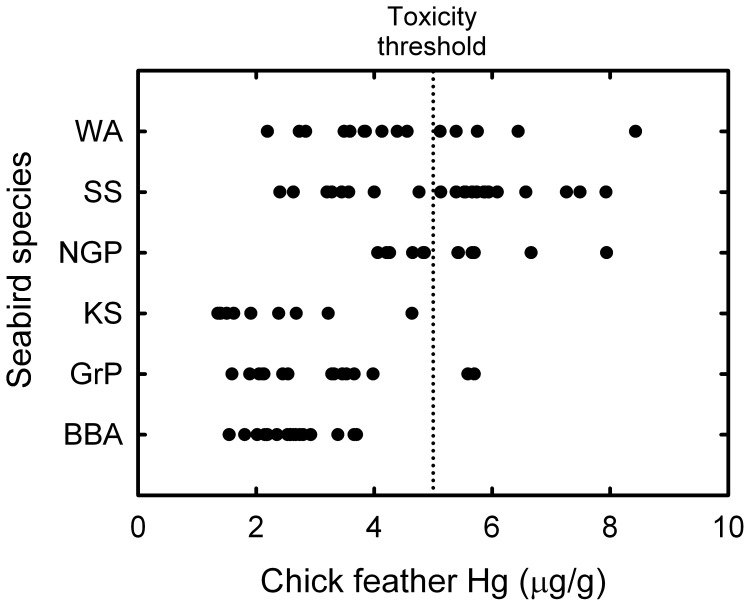
Feather Hg concentrations (µg•g^−1^ dry mass) of individual chicks from the six most contaminated species from the Kerguelen seabird community. See text for toxicity threshold and Table 1 for species abbreviations. BBA illustrates the most contaminated species of the assemblage with all individual values being below the threshold value.

One of the key problems in environmental toxicology is to interpret the impact of observed contaminant concentrations. In general, Hg levels of 1.5–18 µg•g^−1^ dry mass in eggs are sufficient to cause decreased egg mass, embryo malformations, lower hatchability, decreased chick growth, and lowered chick survival [42]. The threshold levels in feathers of negative effects to chicks is currently unknown, but laboratory and field studies on adult birds indicate that feather Hg concentrations of 2.4 to 40 µg•g^−1^ are associated with adverse effects, with the commonest used toxicity threshold being 5 µg•g^−1^ [42], [43]. Feather Hg concentrations of most Kerguelen seabirds are below this threshold, but some individuals from four species showed higher levels. Namely, 64%, 50%, 33% and 13% of chicks of subantarctic skuas, northern giant petrels, wandering albatrosses and grey petrels, respectively, exceeded the threshold value and were thus potentially threatened by Hg (Fig. 3). However, those levels are difficult to interpret because chicks showed no observed obvious ill effects. Moreover, seabirds cope efficiently with high Hg concentrations in their prey through efficient detoxification processes and, hence, they are expected to have higher toxicity thresholds than terrestrial birds [36]. Nonetheless, such comparisons are invaluable to use seabirds as sentinels of ecosystem health, because it provides ways to identify not only the species the more at risk, but also the species that would be useful as bio-indicators [10].

### Feather Hg Concentrations, Diet and Trophic Levels

As expected, the overall statistical analysis indicated a δ^15^N effect on feather Hg concentrations within the Kerguelen seabird community. The positive correlation verifies our hypothesis stating that Hg concentration should increase with trophic level, because δ^15^N is a proxy of consumers’ trophic position [44]. Noticeably, the relationship was partially hindered by pooling species that forage in distinct habitats (neritic vs. oceanic and benthic vs. pelagic) marked by different isotopic baselines. Within this context, the positive correlation between Hg and δ^15^N is particularly relevant. Indeed, the relationship was even stronger when looking at oceanic seabirds only (Fig. 2). The feather δ^15^N values of the 12 oceanic species ranged from 8.8‰ (the crustacean-eater South Georgian diving petrel) to 14.2‰ (the squid-eater wandering albatross), which, assuming a trophic enrichment factor of 2.7‰ for carnivorous organisms [45], corresponds to ∼3 trophic levels. The corresponding feather Hg values indicated an 86-fold Hg enrichment within the oceanic seabird assemblage. Accordingly, Hg contents of pelagic organisms from the Southern Ocean (including Kerguelen Islands) increase in the order crustaceans <fish ≤ squids < seabirds [9], [33], [46]. Hence, while patterns of Hg accumulation in food webs of the open ocean are largely unknown [6], the present work highlights the occurrence of efficient Hg biomagnification processes in subantarctic waters of the southern Indian Ocean that merit further oceanographic investigations.

The subantarctic skua was clearly an outlier species within the Kerguelen seabird assemblage, with chick Hg concentration being high when compared to feather δ^15^N, and, to a lesser extent, δ^13^C values (Table 2). At the study site, adult skuas forage on land where they feed their chicks almost exclusively with small seabirds, mainly blue petrels [47]. Feather δ^15^N value of skua chicks agrees with a blue petrel-based diet, being 2.7‰ ^15^N-enriched when compared to blood of their prey (author’s unpublished data). The skua Hg content is also in agreement with feeding on blue petrels, because petrel muscle contains disproportionately more total Hg than fish and crustaceans [9], [46]. High petrel Hg contamination is likely to result from the combination of two life-history traits of the species: (i) blue petrels are long-lived animals [48] and are thus prone to Hg bioaccumulation over the long-term, and (ii) they prey on crustaceans and mesopelagic fish [49], [50], with the latter containing high Hg concentrations [46], [51]. The trophic explanation of the high Hg levels of subantarctic skua chicks therefore suggests that seabirds feeding on other seabirds are at risk to accumulate critical pollutant loads. Indeed, Scottish great skuas feeding predominantly on other seabirds contain more Hg than those feeding on fish [52], [53], but with no negative effects on breeding performance and survival [54].

### Feather Hg Concentrations and Foraging Habitats

Univariate analysis indicated no δ^13^C effects on feather Hg concentrations within the Kerguelen seabird community, and, in multivariate analysis, species and δ^15^N produced the best model. This result seems to contradict our hypothesis that foraging habitat should play an important role in shaping seabird Hg contamination. However, the large range of seabird δ^13^C values indicates that, again, species that forage within different isoscapes were pooled, thus resulting in a confounding effect [15] for the interpretation of feather Hg concentrations. For example, the five seabirds with δ^13^C values ≥ **−**18‰ were all neritic species feeding either along the shoreline (the kelp gull) or on pelagic (the southern rockhopper penguin and common diving petrel) or benthic prey (the gentoo penguin and Kerguelen shag) (Table 1). The relatively high feather Hg concentrations in feathers of the two latter species are in agreement with the Me-Hg-enrichment of coastal benthic areas [6]. Noticeably, the two species feeding in the closed sea (the southern rockhopper penguin and common diving petrel) and the sibling species foraging in the open sea (the macaroni penguin and South Georgian diving petrel, respectively) have low feather Hg concentrations. Such low levels can be related to a crustacean-based diet and suggest that no significant inshore/offshore gradient occurs in the availability of Me-Hg in pelagic waters surrounding the Kerguelen Islands.

At a larger spatial scale, the importance of foraging habitat is exemplified by the positive correlation between feather Hg concentrations and δ^13^C values in oceanic seabirds. Taken into account the latitudinal δ^13^C gradient within the Southern Ocean [15], [16], the relationship indicates that species foraging in cold waters south of Kerguelen Islands are less prone to be contaminated than species feeding in northern warmer waters. This finding does not fit well with the only study on Hg speciation in the Southern Ocean showing higher Me-Hg concentrations in Antarctic than in subantarctic and subtropical waters [13]. This mismatch reinforces the need to better document the bioavailability of Me-Hg in the main oceanic and neritic water masses and to determine the levels of Hg contamination of seabirds breeding in the north (e.g. Amsterdam Islands) and south (e.g. Adélie Land) of the Kerguelen Archipelago to confirm or not this latitudinal trend both at the bottom and at the top of the marine trophic webs.

### Conclusions

The pattern of Hg contamination of Kerguelen seabirds is remarkable due to its wide range of values, but the circumpolar annular structure of the Southern Ocean [55] suggests it may be generalized to other subantarctic localities. The source of Hg in subantarctic waters is still poorly known, but it ultimately derives mostly from anthropogenic contamination, because (i) human activities have increased emissions to the atmosphere by approximately a factor of 3, and (ii) atmospheric deposition is the dominant input term in the world ocean [6], including the Southern Ocean [13]. Since global Hg emissions will increase in the future, Hg contamination will increase as well in remote areas [56]. Hence, Kerguelen Archipelago, together with other isolated islands located far away from anthropogenic sources, can be considered as ideal study sites to monitor the temporal trend of global Hg contamination. Our study allows selecting chicks of some seabirds as sentinels of environmental pollution according to their high Hg concentrations with relatively low variances and to their contrasted foraging ecology. Three representative species are the gentoo penguin (benthic neritic forager), black-browed albatross (pelagic neritic forager) and light-mantled sooty albatross (southern oceanic forager). Despite its larger variance in Hg concentrations, the wandering albatross (northern oceanic forager) must also be included, because this iconic seabird is known to be among the most Hg contaminated vertebrate species [36], [57].
